# Does clinical ethics need a Land Ethic?

**DOI:** 10.1007/s11019-019-09890-x

**Published:** 2019-04-17

**Authors:** Alistair Wardrope

**Affiliations:** 1 University of Sheffield, 30 Regent Street, Sheffield, S1 4DA UK; 2 The Rotherham NHS Foundation Trust, Moorgate Road, Rotherham, S60 2UD UK

**Keywords:** Land ethic, Environmental ethics, Antimicrobial resistance, Holism, Climate change, Environmental health

## Abstract

A clinical ethics fit for the Anthropocene—our current geological era in which human activity is the primary determinant of environmental change—needs to incorporate environmental ethics to be fit for clinical practice. Conservationist Aldo Leopold’s essay ‘The Land Ethic’ is probably the most widely-cited source in environmental philosophy; but Leopold’s work, and environmental ethics generally, has made little impression on clinical ethics. The Land Ethic holds that “A thing is right when it tends to preserve the integrity, stability, and beauty of the biotic community. It is wrong when it tends otherwise.” I argue that a Land Ethic helps to re-frame problems in clinical ethics that more common philosophical approaches struggle to handle, and that it can be incorporated into clinical ethics without succumbing to “environmental fascism”. I motivate viewing problems in clinical ethics from the perspective of the ‘integrity of the biotic community’, then illustrate how this perspective can offer guidance where more commonly-invoked theories—such as consequentialism and Kantian-inspired approaches—struggle, using antimicrobial resistance in nosocomial infection as a case study. The Land Ethic equips us to understand human values as arising within and inseparable from a social-ecological context, and by treating communities (both human and biotic) as valuable in themselves rather than just through the aggregate welfare of their individual participants, we can avoid problems with the ‘repugnant conclusion’ and utility monster that plague utilitarian accounts.

## Introduction

A thing is right when it tends to preserve the integrity, stability, and beauty of the biotic community. It is wrong when it tends otherwise. (Leopold 1949, pp. 224–25)

The ‘Land Ethic’, a brief essay in conservationist Aldo Leopold’s 1949 collection a Sand County Almanac, is perhaps the most widely-cited work in environmental ethics. But the Land Ethic, and Leopold’s work more generally, have received little attention in biomedical ethics; the ‘global bioethics’ of Van Rensselaer Potter (1988) represents the only real attempt to integrate Leopold’s work, but Potter’s focus was overwhelmingly on public and population health issues. Neither his nor Leopold’s contributions have made a significant impression on clinical ethics.

In this paper, I provide a brief introduction to Leopold’s Land Ethic, and a justification of why clinical ethics should incorporate a Land Ethic. The Land Ethic departs significantly from dominant theoretical approaches in clinical ethics by moving from an exclusive anthropocentrism focused on the individual, to a holistic, biocentric perspective. I seek to illustrate how some of the challenges facing clinical ethics in the Anthropocene—our current geological age defined by human influences on earth systems—necessitate this shift in perspective, using the example of efforts to combat antimicrobial resistance as a case study.

## Leopold’s method

There are two spiritual dangers in not owning a farm. One is the danger of supposing that breakfast comes from the grocery, and the other that heat comes from the furnace. (Leopold 1949, p. 6)

‘The Land Ethic’ is not argued in conventional philosophical style. It contains just four references: three are to classic sources of moral guidance in the Western tradition—the Bible, Homer’s Odyssey, and the Golden Rule; the fourth is to the 1937 Soil Conservation District Law of the Wisconsin State Legislature. What one finds in their place is something as notable by its absence in much academic ethical argument—an intimate acquaintance with the concrete details of the subject of concern. The majority of A Sand County Almanac is given over to sketches of the world around Leopold’s farm, and the relationship of its denizens to the human community and to each other. In place of formal argumentation, one finds depictions of the felling of an old oak tree killed by lightning damage; the preferences of different woodpeckers for birch or pine; the social habits of woodcocks; and the absurdity of state borders to migratory geese. These accounts are supplemented with Leopold’s aesthetic reaction to such phenomena, and (sparingly) theoretical elaboration that situates these tableaux of wild life within a broader understanding of each organism as a node within a physical system.

That Leopold’s conclusions appear to come less from direct argument than as inference from his personal acquaintance with the land and academic knowledge of its organisation frequently leads critics to allege that the Land Ethic commits the naturalistic fallacy, violating Hume’s illicit inference from what is to what ought to be. One might see further confirmation of this suspicion in Leopold’s apparent identification of two definitions of ethics, the ‘ecological’ (“a limitation on freedom of action in the struggle for existence”) and the ‘philosophical’ (“the differentiation of social from anti-social conduct” (Leopold 1949, p. 202); out of context, this can (and has) lead critics to infer that Leopold offered an evolutionary justification of the Land Ethic (Callicott 1989a). This conclusion, however, fails to appreciate how Leopold’s naturalist observations are linked to his ethics; contrary to Potter’s somewhat dismissive categorisation of them as “romantic passages” that it would be “inappropriate” to relate to the Land Ethic itself (Potter 1988, p. 22), they play a key role in his wider argument.

If Leopold offered an evolutionary sociobiological account of the Land Ethic, then his sketches of the natural world would serve little more than rhetorical flourish. But, as the quotation that opens this section makes clear, Leopold argues throughout that those who fail to engage with nature on its terms do not just miss out on a pleasurable experience—they are in ‘spiritual danger’.^1^ The danger in question is of a failure to cultivate the appropriate kind of moral perception—an inability to see oneself as “plain member and citizen” of the land community (Leopold 1949, p. 203). The quotidian descriptions of Leopold’s life and that of his ecological community serve a pedagogical role in attempting to cultivate this kind of moral perception, to enable us to make the shift in our view of land from “a commodity belonging to us” and toward “a community to which we belong.” (Leopold 1949, p. viii).

Seen in this light, Leopold is less trying to derive an ‘is’ from an ‘ought’ than to motivate the reader to see the world in a way that can help to reveal what ‘ought’ to be. Callicott parses this in terms of Hume’s own response to the is/ought dichotomy, the Sand County Almanac serving as ‘intuition pump’ to stimulate our moral sentiments in response to the plight of other members of the land community in the same fashion as they already respond to the situation of other humans (Callicott 1989a). One can alternatively read his approach in terms of the virtue of attentive moral perception highlighted by writers in the virtue and care ethics traditions. Margaret Olivia Little argues that “the attentiveness necessary to good moral judgment is best ensured […] when we care, not simply about impersonal moral ideals such as justice, but about *people themselves*,”(Little 1995, p. 123); virtue theorists at least as far back as Aristotle, meanwhile, have resisted the notion that virtuous conduct is codifiable, but instead propose that it is learned from how the *phronimos sees* and responds to the world. Similarly, Leopold does not attempt to deduce his Land Ethic from abstract principles because it is best understood from within the perspective of one who sees herself as a member of the land community.

If we follow Leopold’s argumentative strategy, acceptance of the axioms of the Land Ethic relies in part on our coming to see the world as he does; the *Sand County Almanac* is as much a work of moral education as moral philosophy.^2^ However, this does not make a Land Ethic any less respectable than other widely-accepted means of resolving foundational issues in clinical ethics. The ‘common morality’ (Beauchamp and Childress 2013), reflective equilibrium (Daniels 1979) and social-contract (Wynia 2008) theories all similarly rely to some extent on agents’ perception of the moral landscape; Leopold’s work serves to broaden those perceptions. I present further arguments for this perspectival shift below, but first describe in a little more detail what the Land Ethic is.

## What is a Land Ethic?

[A] land ethic changes the role of Homo sapiens from conqueror of the land-community to plain member and citizen of it. It implies respect for his fellow-members, and also respect for the community as such. (Leopold 1949, p. 204)

Though often treated as such, it would be a mistake to make the two sentences quoted at the start of this essay the ‘summary moral maxim’ of the entire theory (Millstein 2018). Leopold does not, however, offer any explicit formulation of the axioms of his ethical theory. By way of exegesis, I wish to draw attention to three key components of the ethic here, that comprise the first three sections of Leopold’s essay: (i) the ‘community concept’ that allows communities as wholes to have intrinsic value; (ii) the ‘ethical sequence’ that situates the value of such community as extending, not replacing, values assigned to individuals; and (iii) the ‘ecological conscience’ that makes the Land Ethic not a code of ethics so much as a way of perceiving the moral landscape.

### The community concept

Bioethical arguments commonly assume that all value ultimately inheres in or accrues to individuals; this individualism is so prevalent that it is rarely even explicitly argued for, rather forming an axiomatic “background constellation of values” (Callahan 2003, p. 498). This is not only the case for those working within the Kantian tradition or the principlist school that enshrines individual autonomy above other values. Even in ‘greater good’ theories like utilitarianism or prioritarianism, the way one determines the ‘greater good’ is *as a function of the good of individuals*. For the utilitarian, the greater good is that which maximises marginal utility—the sum of each individual’s utility. For the prioritarian, it is that which best promotes the good of the least well-off. In neither of these accounts is there space for *irreducible community-level* value—that is, saying that some state of affairs described at a level above the individual is more valuable than another, where that value is not simply determined as a function of the value accruing to individuals within it.

The ‘community concept’ of the Land Ethic questions this assumption; in the above right action is defined in terms of its effects directly on “the biotic community”, rather than its members. While there is some debate over the precise definition of Leopold’s ‘land community’ (Millstein 2018), it makes a fundamental unit of moral concern out of the basic structures of ecological ontology—ecosystems, or food/energy networks. Within such systems, individual organisms can be individuated, but they are defined in terms of the larger structure and constitutively dependent upon it (Callicott 1989b). Invoking the ‘community concept’ allows us to take a step away from individualism toward evaluating states of affairs *holistically*—saying that one picture of the world is better than another evaluated at the level of the overall picture, rather than derivatively by evaluating how each participant in that picture fares within it. This has some important and attractive features for certain notorious difficulties with aggregative, individualistic consequentialist theories, as explored in "Consequentialism" section below.

### The ethical sequence

This holism, in fact, is not just a feature of the Land Ethic, but its most frequently-critiqued feature. It is alleged that the ethic is “holistic with a vengeance” (Callicott 1989b, p. 84), the priority assigned to the biotic community leading to “environmental fascism,” (Regan 2004) and the subjugation of individual interests to the needs of the environment. This allegation, however, misunderstands how Leopold situates the Land Ethic in what he calls ‘the ethical sequence’.

Leopold conceives of the ethical sequence as a gradual extension of the scope of ethical considerations—both in the kinds of person they admit as worthy of moral consideration (as classist, sexist, and racist exclusions from personhood are progressively overcome), and the kinds of relations they govern (from two-party relations between individual persons, to progressively more complex relations between individual and society). While this whiggish view of moral progress is anthropologically and philosophically naïve as a *descriptive* picture of moral practices, its primary function in elucidating Leopold’s ethic is *prescriptive*. He envisions this progress as a series of “accretions”, each step in the sequence not supplanting the last, but extending it. In taking seriously our obligations as citizens of a democratic state or ideas of collective responsibility for societal welfare, we do not thereby downplay the need for ethical conduct in our relationships with those we encounter directly in daily life. In similar fashion, taking the community concept seriously need not blind us to the needs of the individual; the Land Ethic demands that the moral agent show (my emphasis) “respect for his [*sic*] fellow members, *and also* respect for the community as such.” (Leopold 1949, p. 204).

### The ecological conscience

The third component of the Land Ethic has already been introduced in discussing Leopold’s ethical method. He resists the idea that the Land Ethic can be realised through any form of codification of obligations, for two reasons: we do not have a sufficiently full understanding of ecological science to describe exactly what each member of the land community’s obligations should be in order to maintain the community (Leopold 1949, pp. 194–197; 204–205); and imposing obligations to the land community without understanding one’s membership of it results in action according to the letter, but not the spirit, of those obligations, and consequent failure to secure the “integrity, stability, and beauty” of the community (Leopold 1949, pp. 207–209). Leopold argues that the Land Ethic instead requires development of the “ecological conscience”—a way of perceiving the moral landscape that makes our responsibilities to the land community salient. As explored further below, this lends itself to an interpretation of the Land Ethic in the light of virtue ethics—but crucially, where the ends of virtue are not so much the cultivation of individual flourishing, but rather that of the land community of which one is a member.

## The Land Ethic, bioethics, and clinical ethics

There are two organisms whose processes of self-renewal have been subjected to human interference and control. One of these is man himself […] The other is land. (Leopold 1949, p. 194)

### Two kinds of bioethics

The claim made in the introduction that the Land Ethic has received little attention in bioethics is complicated by the multivocal nature of the term ‘bioethics’. The origins of bioethics in twentieth century Anglophone discourse experienced a “bilocated birth” (Reich 1995; Potter 1988, Chap. 4; ten Have 2012; Lee 2017)—one in the work of Van Rensselaer Potter, and another with André Helleger and colleagues at Georgetown University. In Potter’s use of the term, bioethics was to become a discipline that sought to utilise “the amalgamation of ethical values and biological facts” (Potter 1988, p. 71) to promote “acceptable survival” of human society and the natural environment (Potter 1971, 1988). The Georgetown model, meanwhile, has a much narrower scope, focusing chiefly on ethical dilemmas arising in clinical practice, medical research, and from the structure of healthcare institutions and distribution of healthcare-related goods.

In Potter’s most heavily Land Ethic-influenced work, *Global Bioethics*, he identifies the existence of these two kinds of bioethics, dubbing the first ‘ecological bioethics’ and the second ‘medical bioethics’ (Potter 1988, Chap. 4). He also notes that in the first decades after bioethics’ coalescing as a discipline, it was the latter that received overwhelmingly greater attention and came to define the field. Potter was uncompromising in his assessment of our likely fate if the pre-eminence of medical bioethics were permitted to persist unabated; a short-term focus on individual health interests with a neglect of the opportunity costs and ecosystemic implications would condemn us to a future of at best “miserable survival” (Potter 1988, p. 75).

Accusing bioethics of a neglect of the Land Ethic, then, is perhaps a specific instance of the more general problem of bias toward Georgetown-style bioethics and away from Potter’s. Renewed interest in the role of the environment in human health and the existential threats posed by major environmental challenges have led several authors to draw attention to this bias and to attempt to redress it (ten Have 2012; Resnik 2012; Berkes et al. 2012; Verweij and Bovenkerk 2016; Lee 2017; Gribble 2017; Buse et al. 2018). Drawing from the distinct discipline of environmental ethics, and the continued tradition of engaging with environmental health threats as a matter of justice in both the campaigning and theoretical environmental justice movements (Shrader-Frechette 2002; Schlosberg and Collins 2014), these authors have begun to develop environmental health ethics as a proper part of bioethics, giving a more three-dimensional view of the field.

### A three-dimensional view of bioethics

Potter’s subdivision of bioethics into ‘medical bioethics’ and ‘ecological bioethics’ can be thought of instead as an axis along which bioethical discussion may vary—the medical-ecological (or ‘Georgetown-Potter’) axis. The variation along this axis is one of *scope* in subject matter. At one—Georgetown—end, the ethics of life and health is concerned primarily with human health, specifically as shaped by human healthcare institutions. This is the ethics of “resolving practical ethical dilemmas related to clinical care and clinical research” (Lee 2017)—in other words, clinical ethics. At the other—ecological—end lie questions regarding the value of the natural world and our standing within it.

Lee (2017) offers two further axes along which bioethical theorising can vary: the anthropocentric-ecocentric, and individual-systemic axes.^3^ The first of these is the *value-bearing* axis—what sorts of things have morally relevant interests that should guide our deliberations? Humans alone? Sentient creatures, or those who do not have a “subjectively barren form of existence” (Singer 1980)? Or should we practice the “biospherical egalitarianism” (Naess 1973) of Deep Ecology? The second axis is of *scale* (Buse et al. 2018)—the level of individuation or aggregation of entities, and relevant temporal intervals, in terms of which a problem is being considered. In human terms, scale can range from individual persons, through communities and institutions, to societies and the global population. Biologically, we can move from individual organisms to entire ecosystems, right up to planetary boundaries. Temporally, we might look at a problem as an individual, isolated decision-point, or over the course of seasons, years, electoral cycles, generations, or geological eras.

This three-dimensional view of bioethics allows us to position different theoretical approaches relative to one another, highlighting which might be more relevant for different classes of problem, and which areas might remain underexplored. David Resnik’s principlist approach to environmental health ethics (Resnik 2012), for example, engages chiefly with issues of environmental public health policy—these subjects lying somewhere in the middle of the medical-ecological axis—though with his principles of ‘stewardship’ and ‘sustainability’, and by engaging with larger environmental health topics such as climate change, he moves further toward the ecological end. Given his emphasis on policy recommendations and a pragmatic desire to avoid radically revisionist principles, he argues on the anthropocentric-ecocentric axis for an ‘enlightened anthropocentrism’ that assigns some—though lesser—moral value to non-human life, and is agnostic on the question of the intrinsic or extrinsic nature of this value. Lee (2017) makes an explicit case for public health ethics being situated at the middle of the medical-ecological axis and so forming ‘a bridge back to the future’ between these two poles. She proposes that public health ethics encompasses at different times the entirety of the scale axis. Buse, Smith and Silva, however, contend that Lee’s conception of scale is inadequate for properly meeting environmental health challenges insofar as it assumes that different levels of scalar ethical analysis (micro/meso/macro) can be treated more or less independently; they propose instead that the moral significance of nested relationships between these levels require an ethic capable of ‘zooming in’ and ‘zooming out’ to engage with multiple levels simultaneously (Buse et al. 2018). In this respect, their ethic is reminiscent of the “total field view” of Naess’ Deep Ecology (Naess 1973)—though they do not share Naess’ firm ecological and ecocentric focus.

What recent attempts to redress the predominance of medical bioethics share, however, is an attempt to move away from the Georgetown end of the medical-ecological axis, and towards Potter’s. Their objective is to acknowledge the importance of environmental health to bioethics and to take seriously the need for an environmental health ethics. This is an important project for bioethics as a discipline, and bioethicists as professionals. However, it is not the project of this paper. As suggested by the emphasis on “clinical ethics” in the title, I aim to remain focused on medical bioethics—but to move away from the ‘Georgetown model’ by using the Land Ethic to explore the implications for clinical ethics of variation along the anthropocentric-ecocentric and individual-(eco)systemic axes. This, I will argue, is an important project for clinical medicine as a discipline, and clinical practitioners as professionals.

While an undertheorised project, it is not one that has been entirely neglected. Potter himself explicitly addresses some clinical questions in his global bioethics. However, the topics chosen and approach taken are almost exclusively dominated by his Malthusian preoccupation with overpopulation (Potter 1988). Clinical ethics needs more than this, I shall argue, not only because the interactions between clinical practice and the natural world are more numerous and complex than Potter considers, but also because the relationship between population and sustainability is more nuanced than Potter accounts for; as a recent review of the subject puts it, “consumers, rather than people, cause climate change” (Stephenson et al. 2013). Goldberg and Patz, meanwhile, argue the need for a ‘global health ethic’ that is explicitly inspired by the Land Ethic (Goldberg and Patz 2015); however, they deliberately avoid addressing questions of what such an ethic would entail, how it should modify clinical practice, or how it could be cultivated in practitioners. I aim to start to address these questions, after first arguing that they do need answers.

## Why a Land Ethic for clinical ethics?

To motivate the premise that our relationship to the land community is a proper concern also of clinical ethics, I provide both anthropocentric and biocentric justifications.

### Anthropocentric arguments

When the logic of history hungers for bread and we hand out a stone, we are at pains to explain how much the stone resembles bread. (Leopold 1949, p. 210)

The Land Ethic seeks to move our moral perceptions away from an exclusive anthropocentrism; providing anthropocentric justifications for its adoption, then, somewhat misses the point. But pointing out how these stones resemble bread might at least serve to open the mind of the sceptic. An anthropocentric motivation for extending our moral concern to the land community comes from acknowledgement that a failure to do so threatens the health of the patients and populations who are more traditionally considered the focus of clinical ethics.

That human health is intimately entwined with the environment is becoming increasingly apparent as human activity moves the planet beyond its safe operating space. Climate change—potentially “the greatest threat to global health of the twenty first century” (Costello et al. 2009)—already accounts for (at a conservative estimate) an excess 250,000 deaths per year (WHO 2017). Heat-related morbidity in Europe is predicted to increase more than threefold in Southern Europe (Åström et al. 2013). The global distribution of zoonotic infectious diseases is shifting to expose more-susceptible populations to increased risks of disease like malaria and Dengue fever (Caminade et al. 2014; Bouzid et al. 2014). Changing atmospheric CO_2_ levels are altering the nutritional content of staple crops, putting an estimated 138 million more people worldwide at risk of zinc deficiency by 2050 (Myers et al. 2015). Ambient air pollution account for 7.6% of total global mortality in 2015 (Cohen et al. 2017). And so on.

This establishes that our relationship with the land community is a proper concern of those interested in human health, perhaps; but what makes it appropriate for *clinical* ethics? These topics may seem to shift us toward the ecological end of the medical-ecological axis, being the proper focus of public or environmental health ethics in theory, and health policy in practice. To make this assumption, however, is to neglect considerations of scale. To assume a neat division between clinical and public health is to ignore the ‘nested relationships’ (Buse et al. 2018) between what Susan Sherwin calls ‘levels of human organisation’ (Sherwin 2011)—the finer or coarser-grained descriptions of social networks (from individuals, through intimate social groups, via community organisations and businesses to local, national, and international governmental agencies). Dividing these levels permits one to make one the focus of public health, the other of clinical medicine. But as Sherwin notes, the parties of one level also contribute to higher and lower levels, and actions at any given level constrain the options available at other levels. It is the actions of individuals—clinicians included—that cumulatively produce the environmental changes described above that have such damaging effects on the health of others. Thus clinical ethics cannot absolve itself entirely of responsibility for its cumulative, spatially-and temporally-distributed, downstream effects. I explore the ramifications of this point for clinical ethics further in the example of antimicrobial resistance below.

### Biocentric arguments

[We] have learned (I hope) that the conqueror role is eventually self-defeating. Why? Because it is implicit in such a role that the conqueror knows, ex cathedra, just what makes the community clock tick, and just what and who is valuable, and what and who is worthless, in community life. It always turns out that he knows neither, and this is why his conquests eventually defeat themselves. (Leopold 1949, p. 204)

While we can motivate consideration of the land community from a purely anthropocentric perspective, this is still to position ourselves as apart from that community. Properly embracing the Land Ethic requires adopting the perspective of a member amongst others—the shift in roles from ‘conqueror’ to ‘citizen’ in Leopold’s metaphor. Here I provide two considerations to motivate this shift: the reality of the impact of clinical practice on the environment; and the ‘self-defeating’ nature of the conqueror role.

In the previous section, we reviewed some of the extensive evidence of the impact of the environment on human health. It is the nature of ecological interdependencies that these relationships are bidirectional, and as such it is hardly surprising to find that healthcare is a major driver of global environmental change. This is particularly the case in the resource-intensive models of healthcare practised in industrialised Western nations; the health sector in the USA accounts for an estimated 8% of the country’s entire greenhouse gas footprint (Chung and Meltzer 2009), while the NHS in England’s footprint in 2015 was 22.8MtCO_2_e (just under 5% of the UK total) (NHS SDU 2016). This is largely driven by routine clinical practice (for example 21% of the total NHS footprint comes from pharmaceutical prescriptions, a further 11% from medical instruments (NHS SDU 2013).) Pharmaceuticals also affect the local environment both in manufacture and use—for example, environmental accumulations of diclofenac have been associated with declining vulture populations due to renal failure (Oaks et al. 2004), with concerns also raised about the effects of environmental accumulations of drugs from anti-epileptic agents to synthetic steroids (Al Aukidy et al. 2014). If clinical workers consider themselves at all members of the land community, then the aggressive exploitation of this community by current models of clinical practice demands that we urgently rethink how we approach that practice.^4^

The reason I suggest that a pure anthropocentrism is self-defeating is that if we consider ourselves as divorced from the biotic community then we fail to appreciate what it is that makes us individuals. As those critical of the ‘atomism’ of liberal individualism argue, our ‘authentic’ identities are socially constituted; we cannot understand or even formulate a set of values, ideals, or desires that constitute reasons for action except in dialogue with our cultures, communities, and personal relationships (Taylor 1991; Mackenzie and Stoljar 2000). As Jonathan Beever and Nicolae Morar argue, this interdependence goes beyond the human community alone. They highlight the extent to which characteristic features of human culture (such as diet) both depend upon and shape the commensal microbial community (‘microbiome’) of members of that culture, and how the consequences of our actions—and even decision-making processes themselves—may be extensively dependent upon gut microbiota (Beever and Morar 2016). They argue that, as a consequence, “[m]icrobial ecology plays a central role not only in understanding the individual as a community, but also in recognising the ways that agency and autonomy are … impacted by microbial interactions.” Until we view ourselves as members of the land community, we cannot understand ourselves in the ways that an anthropocentric, individualistic ethic would require. To create a clinical ethic that does not undermine itself in this way might require that we embrace the community concept.

## Land Ethics and biomedical ethics

To understand better the theoretical and practical implications of the Land Ethic for clinical ethics, it may help to situate the Land Ethic amongst more familiar approaches. The attempt to do so here is, for reasons of space, incomplete and allusive; the comparisons here serve chiefly to highlight some implications of the Land Ethic that prove appealing for resolving established difficulties with some of the dominant ethical apparatus employed in clinical ethics.

### Kantian ethics

An oak is no respecter of persons. (Leopold 1949, p. 9)

Those inspired by Kant find the normative force of our obligations to arise from the recognition of another as a rational agent, one capable of sharing and acting upon reasons. But the majority of the citizens of the land community lack this capacity, even with the most generous understandings of reason or will—an oak cannot respect us, so we should not respect the oak. While some have attempted to argue that the evolutionarily-defined teleology of natural systems grant them an ‘autonomy’ that could be subject to Kantian respect (Gillroy 1998), such a loose understanding of autonomy cannot function as the premise in Kant’s argument that we have a duty to respect the autonomous wills of others (Korsgaard 1996, 2004). In this argument, it is the need to reflect upon and act upon reasons that is worthy of respect; thus Kant rules out straight away the idea of duties owed directly to (the majority of) non-human entities or to ‘communities’. While some seek to reconstruct a Kantian environmental ethics (Svoboda 2012; Korsgaard 2004), duties toward the non-human biotic community in these approaches are derivative of the ultimate perfect duties that individual human agents hold toward one another. The wrong of threatening sustainable ecosystems comes in failing to acknowledge the good of our own animal nature (Korsgaard 2004), or in lessening one’s own moral purity (Svoboda 2012). Kantian ethics is, to this extent, definitionally both individualistic and anthropocentric, and struggles to embrace the community concept that allows us to see entities beyond individuals as bearers of intrinsic value.

### Consequentialism

It did not occur to the Governor that there might be more than one definition of what is good, and even of what is business. It did not occur to him that while the courts were writing one definition of goodness in the law books, fires were writing quite another one on the face of the land. (Leopold 1949, pp. 10–11)

To the extent that it evaluates right action (at least in part) in terms of its contribution to the “integrity, stability, and beauty of the biotic community”, the Land Ethic is a consequentialist one. But by taking that community concept as of fundamental value—rather than a function of the good of its individual members—it differs from the dominant consequentialist methodology in bioethics (and indeed throughout most of the practical applications of consequentialist theory), which always picture good as accruing to individuals alone (whether in the form of preferences, utils, QALYs or otherwise). By introducing ‘stability’—best parsed, as Millstein argues, in terms of ‘sustainability’ or ‘resilience’ *(*Millstein 2018; Berkes et al. 2012*)*, it also presents a conspicuously diachronic picture of morally relevant consequences.

Evaluating states of affairs holistically and diachronically can help to resolve some notorious paradoxes of purely individualistic consequentialist theory—notably Nozick’s ‘utility monster’ and Parfit’s ‘repugnant conclusion’. These are both problems that arise from determining best consequences as the sum of the value accruing to all individuals in a given state of affairs. The utility monster is an individual who gets vastly more value than everyone else for a given allotment of resources, and as such it will always maximise aggregate welfare to allot resources to this individual, despite the resulting extreme inequality (Nozick 1974). The repugnant conclusion, also known as the mere addition paradox, is that we can maximise welfare by reducing each individual’s welfare but adding many more individuals to the world (a world of 100 people each with 100 utils of welfare will have lower aggregate welfare than a world of 10 billion with just 0.01 utils apiece) (Parfit 1984). But the worlds of both utility monster and repugnant conclusion seem intuitively worse than the alternatives, hence their being levelled as arguments against consequentialist theories.

Both the utility monster and the repugnant conclusion are results of individualistic consequentialist theory. By this I do not mean a consequentialism that considers only measures targeted at the individual, but one that evaluates states of affairs as a linear function of the good of all individuals within that state of affairs.^5^ That is, Utility *U* is the sum of individual utilities *u*_*i*_ across all *N* morally-relevant individuals *i* (potentially weighted with weights *a*_*i*_):

1 $$\begin{array}{*{20}{c}} {{\text{U}}=~\mathop \sum \limits_{{\text{i}}}^{{\text{N}}} {{\text{a}}_{\text{i}}}{{\text{u}}_{\text{i}}}} \end{array}$$

The utility monster describes the situation where, for some *k, u*_*k*_ >>> *u*_*i*_, for all *i* ≠ *k*; in this case (for non-zero *a*_*k*_) *U* ≈ *u*_*k*_. The repugnant conclusion, meanwhile, observes that, provided the *a*_*i*_ and *u*_*i*_ are non-negative (that is, there is no individual whose suffering actively makes the world a better place; and everyone is at least better off than they would be if they did not exist), we can arbitrarily increase U simply by increasing N.

A holistic consequentialism, however, is not required to evaluate U according to Eq. (1), and so is not forced to accept either conclusion. At the most basic level, a community-level consequentialism can simply assert by stipulation that utility-monster and repugnant-conclusion worlds are less valuable than their alternatives. However, the Land Ethic provides us with the resources to say more than this, and to understand why they are less valuable. When we consider individuals as citizens of the land community, their good becomes intrinsically relational (Naess 1973); my wellbeing cannot be understood without reference to that of the rest of the land community, and as such Eq. (1) does not hold. Furthermore, understanding the good at the level of the biotic community helps us identify at least part of what it is that makes the world of the utility monster and the repugnant conclusion so unappealing. The good of the Land Ethic centrally involves the resilience, or long-term stability, of the biotic community. Radically shifting the balance of ecosystems in favour of single individuals or types of organism, however—as human attempts at landscape engineering show us to our cost—is antithetical to such resilience (as, for instance, the ever-greater resource demands and diminishing returns of monoculture demonstrates). Neither the human nor the land community could maintain the utility monster’s exploitation of social and natural resources. Similarly, while abstract communities might maximise their aggregate welfare by indefinite mere addition, biotic communities have a finite carrying capacity. Adding to these communities beyond this capacity does not just diminish the welfare of those already present, it undermines the ability of the community to continue at all in anything resembling its previous incarnation. Thus the repugnant conclusion fails to secure the good of the biotic community, in fact presenting a significant threat to it.

### Virtue ethics

No important change in ethics was ever accomplished without an internal change in our intellectual emphasis, loyalties, affections, and convictions. (Leopold 1949, pp. 209–210)

From the interpretation of Leopold’s ethical method above, it is probably evident that I hold there to be a great deal of affinity between virtue ethics and the Land Ethic. Realisation of the Land Ethic comes first from a shift in who we are and how we see the world and our relation to it; it is only once we understand ourselves as citizens of the land community that we can act in accordance with the ethic. The question here, though, is to what extent the virtues of the Land Ethic can be seen as fit virtues for environmental ethics.

For virtue ethics to be morally compelling, it needs some grounds for its normative force. This may come from some other ethical theory—as with those who see virtue ethics as an instrumental means of realising consequentialist goals (Driver 2001; Jamieson 2007). Or it can come from a perception of virtue as the necessary means of realising some end that is good in itself—Eudaimonia, in Aristotelian terminology. As I have already suggested, Leopold’s concern for the resilience of ecosystems indicates an openness to consequentialist considerations that could be read as making the Land Ethic an instrumental VE. But crucially, Leopold appears to view the moral significance of the “integrity, stability, and beauty of the biotic community” as flowing naturally from our recognition of our position within that community, grounding the normative force of the ethic in pursuing the Eudaimonia appropriate to members of the land community.

For situating the Land Ethic in clinical ethics, then, we must consider to what extent the characteristic activities of a member of the land community can be reconciled with those of a member of the clinical professions. The most developed account of a virtue ethics for the clinical professions^6^ is that found in the writings of Edmund Pellegrino and his co-authors, who ground their analysis of virtue in the characteristic activity of clinical healthcare—the “right and good healing action for a particular human being” (Pellegrino 2006). Viewing clinical practice in this way immediately presents a challenge with any attempt to integrate the Land Ethic into the virtuous clinician’s perspective; Pellegrino presents the foundation of medical ethics as arising from a relationship between individuals, and the clinician’s responsibilities as consequently being owed to their individual patients. Indeed, Pellegrino makes a virtue of the consequent partiality of the clinician’s responsibilities to particular individuals, seeing the “engrossment” (Noddings 1984) in the needs of another, to the exclusion of competing demands, as requisite for achieving the ends of medicine. It is a challenge to cultivate a ‘deep-focus’ moral perception that sees another at one and the same time as an individual patient to whose wellbeing one is wholly committed, and also a node amongst many in the vast network of the biotic community (Buse et al. 2018).

Yet at times both Pellegrino and Leopold seem to demand precisely this simultaneous moral appreciation of the individual and the community. As described above, Leopold did not see the Land Ethic as supplanting responsibilities between individuals, but rather extending our moral perspective to encompass the non-human world as well. Pellegrino, meanwhile, appears to be reaching toward the shift in moral perception demanded by the Land Ethic when he writes that:

In earlier eras the remote effects of medical acts were of little concern, and the rights of the individual patient could be the exclusive and absolute base of the physician’s actions. Today, the growing interdependence of all humans and the effectiveness of medical techniques have drastically altered the simplistic arrangements of traditional ethics. The aggregate effects of individual medical acts have already changed the ecology of man (Pellegrino 1973, p. 138).

When the welfare of the individual patient can be considered in isolation from their social/material/environmental context, then working towards the ‘right and good healing action’ may be achieved purely through an ‘engrossment’ in the individual patient. But if we move away from the atomistic understanding of individuals criticised above toward an expanded moral perspective that understands the individual as constitutively dependent upon their relations to their community, then taking seriously the moral demands of that community becomes an integral part of working toward the ends of clinical practice. While Pellegrino here focuses purely on the human community, given the interdependence between human and land described above, his arguments naturally extend to the land community. Thus, difficult as it may be, virtuous clinical practice on Pellegrino’s model seems to require that we cultivate the kind of moral perception that can focus on a person both as an individual with a medical need, and as a citizen of the land community. The intention of the next section is to consider how this deep-focus perspective can contribute to clinical ethics.

## The Land Ethic and clinical ethics: antimicrobial resistance and nosocomial infection

The practices we now call conservation are, to a large extent, local alleviations of biotic pain. They are necessary, but they must not be confused with cures. The art of land doctoring is being practiced with vigor, but the science of land health is yet to be born. (Leopold 1949, pp. 195–196)

Antimicrobial resistance (AMR) is the process by which pathogenic organisms (e.g. bacteria, viruses, fungi, protozoans) acquire traits that make antimicrobial drug treatments ineffective against them. While some antimicrobial traits (e.g. β-lactamase, an enzyme that renders bacteria insensitive to penicillins and related antibiotics) long predate the human use of antibiotics, anthropogenic selection pressures have vastly accelerated the development and spread of AMR (Holmes et al. 2016). That AMR poses a major threat to human health globally is not widely disputed; the 2016 Review on Antimicrobial Resistance estimated that by 2050 a potential 10 million further lives per year would be at risk due to drug-resistant infections (O’Neill 2016). The mechanisms responsible for AMR are diverse and likely differ between pathogens, but include: inappropriate or excessive use of antimicrobials in human and veterinary health; agricultural misuse of antibiotics e.g. as animal growth promoters; environmental pollution leading to accumulation in soils and water resources; and trade and travel of humans and biota nationally and internationally (Holmes et al. 2016).

The challenge of AMR is not predominantly ethical—the widespread acceptance of AMR as a pressing issue for clinical and public health, and the broad menu of policy and practice options already endorsed, shows that it is already taken seriously as a major challenge to human and environmental health. Furthermore, the rise of the ‘One Health’ movement that explicitly seeks to address human, animal, and environmental health simultaneously suggests an appreciation of the moral importance of the biotic community in addressing human health issues (Robinson et al. 2016). In this case, health workers’ moral sensitivity seems to have developed beyond the theoretical resources bioethics has to offer, because it is not so easy to appreciate how much help these resources can offer. I do not mean to suggest here either that adoption of a Land Ethic will help to resolve the threat of AMR. My intention here is more humble—to suggest that the conceptual tools of the Land Ethic, in particular the development of an ‘ecological conscience’ and understanding our moral responsibilities in light of the ‘community concept’, can play a motivational role in helping clinical workers play their part in confronting this threat.

The ethical challenges of AMR resemble those of other collective-action environmental problems such as climate change. The “perfect moral storm” (Gardiner 2006) that makes climate change an ethical conundrum is present also with AMR. The harms of AMR are cumulative, collective, and spatially and temporally dispersed, so in any individual act contributing to AMR it is both difficult to determine who is harmed, and the individual contribution is so small to the cumulative effect as to be negligible. This dispersion of cause and effect and fragmentation of agency is difficult to manage with institutions and ethical codes that see harms as arising from discrete acts of individual agents perpetrated against others. Problems having these features are notoriously difficult to address using the resources of individualistic, anthropocentric ethical theories (Gardiner 2006; Jamieson 2007).

Consider, for example, the consequentialist clinician trying to reason through how their antibiotic prescribing practices may contribute to AMR. They are one amongst many: even if they made all future prescriptions in a way that exerted no selective pressure for resistance mechanisms; even if they prescribed only responsibly-manufactured products; even if they ensured that no excreted active metabolites passed into waste water, it would make little appreciable difference to the overall development of the problem. They struggle to determine who exactly would be harmed by AMR anyway, certainly in comparison to the possibility of failing to treat an active bacterial infection in the patient in front of them. And the norms and legal regulations surrounding their profession make it clear that none would blame them for the odd unnecessary prescription, whereas lawsuits or professional sanction could quickly follow from undertreating a bacterial infection (even one that on clinical appearances was initially indistinguishable from a cold).

The Kantian will fare little better. Who is the person being treated solely as a means when antibiotic contamination of waste water disrupts soil microbiota or fish reproduction? These can both have downstream deleterious consequences for health of human communities, but it is hard to individuate identifiable persons who are being wronged in these situations. Turning instead to agents’ imperfect duties to improve their own moral perfection is of little use here either, since such imperfect duties can always be overridden by acting in other ways to achieve that end, and clinicians would no doubt argue that focusing on the health of their individual patients is a better means of working towards such a goal. Trying to parse their responsibilities in terms of discrete duties poses a further problem for the Kantian. Leopold rejects attempts to codify environmental obligations in explicit rules or policies because we do not understand well enough the full interdependencies between members of the biotic community. This is demonstrated well in one-size-fits-all attempts to tackle AMR with enforcement of antibiotic prescribing policies; there is good evidence that such enforcement increases adherence to these policies, but not that it reduces rates of antibiotic misuse-associated infection (Davey et al. 2017). This is unsurprising if we consider that the mechanisms of AMR are drug-, bug- and host-dependent (Holmes et al. 2016). For any given combination of those the development or spread of resistance may have less to do with antibiotic choice than: how those antibiotics were made and their waste managed; prevalent diets and the food economies that shape them; or patterns of local and international travel involving a given region. It is for this reason in part that Leopold argues for the ‘ecological conscience’ that does not merely act in accordance with environmental regulations, but evaluates each action in terms of its potential perturbations of the biotic community.

Both individualistic consequentialism and Kantian ethics view AMR as a wrong insofar as it threatens the health of current and future humans. But AMR is only partially, and derivatively, a problem for those humans. More directly, it represents an anthropogenic disruption of ecosystems that, through their injudicious and exploitative perturbation, threatens their long-term resilience, in ways deleterious to the health of many members of the biotic community (humans included). Allowing only potential future humans to feature in our moral deliberation allows more room for the kinds of self-deceptive strategies that result in the ‘moral corruption’ that helps us get around addressing the moral urgency of collective action problems (Gardiner 2006). The Land Ethic, by contrast, forces us to take seriously responsibilities directly to the biotic community as a whole, seeing how our actions serve to perturb that community in the present in ways that reduce its robustness to future threats.

The Land Ethic also asks us to view ourselves not just as having responsibilities to the biotic community, but as citizens of that community, unable to exist apart from it. From this perspective, it is less easy to separate ‘personal’ from ‘professional’ responsibilities, or to stratify neat levels of human organisation which might allow clinicians to consider certain problems issues for ‘public health’ and thus not their problem. AMR demonstrates the need for this shift in perspectives.

Amongst the menu of policy responses required for tackling AMR, clinicians might think that their proper concern relates to the treatments provided to individual patients, while health and agricultural policy should deal with issues like agricultural antibiotic use, or globalisation of resistance through trade and travel. But from the ecological perspective, responsibilities for engaging with these threats are not distributed equally—our different positions within the ecological network mean our actions will perturb it in different ways. The nature of front-line clinical care means that clinicians are far more likely to encounter people with chronic polymicrobial infection, who are immunosuppressed, or otherwise provide ideal environments for intra-individual development or horizontal transfer of AMR (Holmes et al. 2016). The way we structure clinical services can increase or limit demand for intra- and inter-hospital patient transfers that then facilitate further spread of resistant pathogens to new vulnerable populations (Donker et al. 2012). We have known at least since the days of Ignaaz Semmelweis that clinicians are ideally situated to be the vectors of infectious disease; since the potential of clinicians to act as vectors of AMR may depend on their diet (Kluytmans et al. 2013) or where they go for their holidays (Paltansing et al. 2013), we cannot carve off these issues from the proper concerns of clinical ethics.

A Land Ethic-based approach to AMR for clinicians, therefore, may help to provide a supportive normative rationale for non-prescription of antibiotics in situations where the clinicians feels they are unlikely to be of benefit. Even in situations of clinical equipoise, it would encourage use of strategies such as delayed prescriptions, which can significantly reduce antibiotic usage without adversely affecting patient outcomes (Spurling et al. 2017). The Land Ethic does not simply provide an alternative answer to the question of antibiotic prescribing than other ethical theories, however; it encourages clinicians to consider their role in AMR as going far beyond their prescribing powers. It also motivates them to: minimise unnecessary patient or staff transfers to prevent spread of resistant pathogens; take extra infection control precautions when working in multiple healthcare settings—or travelling elsewhere—to avoid propagation of resistant organisms; even to consider the influence of their diet on AMR, for example by avoiding intensively-reared meat. It requires that they look at themselves not just as professionals, but as organisms in an ecological network—and thus as capable of serving also as disease vector and culture medium.

The Land Ethic asks clinicians to consider AMR as a problem for the ecosystem now and not just humans in the future (thus avoids e.g. pinning all our hopes on developments of new drugs) and requires that they acknowledge their own status as citizens of the biotic community (and thus that all their actions, whether ‘clinical’ or not, have consequences that percolate through a network of ecological interdependences in ways that may exacerbate or ameliorate AMR). It is not directly the solution to AMR; but its wider acceptance may help to close the gap between what we know needs to be done, and our motivations to do it.

## Conclusion: a Land Ethic for clinical ethics

Our present problem is one of attitudes and implements. We are remodelling the Alhambra with a steam-shovel. We shall hardly relinquish the steam-shovel, which after all has many good points, but we are in need of gentler and more objective criteria for its successful use. (Leopold 1949, p. 226)

While it is not my intent to codify the Land Ethic into a set of guidelines for clinicians (nor, as discussed above, would Leopold consider that an achievable goal), I will close by suggesting how a Land Ethic might help to shape clinical practice fit for the Anthropocene.

Returning to the three-dimensional view of bioethics in “The Land Ethic, bioethics, and clinical ethics” section, the Land Ethic seeks to cultivate a ‘deep-focus’ or ‘total field’ view of the anthropocentric-ecocentric and individual-systemic axes (Naess 1973; Buse et al. 2018). As explored in the case of AMR above, this requires that clinicians do not isolate their individual practices from the social and environmental processes that both influence and are influenced by their actions. This will take different forms for health workers in different roles. A nephrologist might seek to: empower patient self-management to prevent progression of kidney disease to the hugely resource-intensive dialysis-dependent stage; simultaneously shape public policy in an advocacy role by drawing associations between environmentally damaging industrial agriculture and the dietary drivers of kidney disease; and reduce the resource burden of dialysis by e.g. recycling dialyser reject water and increasing dialyser reuse (Connor et al. 2010; Connor and Mortimer 2010). A respiratory physician might: switch to prescribing inhalers that do not contain greenhouse gas propellants; but also limit their own contribution to an unhealthy environment by walking or cycling rather than using polluting private motorised transport; and advocate for workplace policies that enable other health workers and patients alike to do similarly (British Thoracic Society 2019). A general (family) practice might establish a community farm on its land and support people they encounter as patients in establishing a food co-operative (Buck 2016). And so on for health workers in other contexts.

Leopold’s Land Ethic affords a complementary lens through which to view clinical practice, one that brings into relief issues obscured in other, more prevalent, theoretical foundations for medical ethics. The purpose of this article is not to argue that the Land Ethic should supplant more-established theoretical approaches, but rather that it can be usefully employed to address important but heretofore under-acknowledged challenges.

## Footnotes

1 It is not even enough for Leopold simply to be in wild and green space, as evident from his disdain for “the motorized ant who swarms the continents before learning to see his own back yard” (Leopold 1949, p. 176).

2 The rejection of neat distinction between arguments for and education in morality is common to virtue-ethical theories too (Annas 2011).

3 Lee explicitly relates these axes only to environmental ethics, but they apply more broadly. The first of these she labels the ‘egocentric-ecocentric axis’; I prefer the term ‘anthropocentric’ since at one extreme of this axis lies the position that only human interests are morally significant—i.e. anthropocentrism, not egoism.

4 Nor is it enough for the clinician to dismiss these concerns as necessary evils in the laudable pursuit of more direct clinical objectives like patient health, for there is good reason to believe much of these effects are not *necessary*. The NHS, for example, has managed to achieve a 10% reduction in greenhouse gas emissions from 2007 to 2015 despite an 18% increase in clinical activity over the same period (NHS SDU 2016), while less resource-intensive models of care used in less-industrialised nations may provide still greater benefits (Syed et al. 2012).

5 One could make, for example, an individualistic argument (in this sense) for a collective intervention such as publicly-funded healthcare if one gave as justification the aggregate gain of DALYs from such an intervention.

6 At least for doctors, since Pellegrino focuses on medicine rather than other clinical professions.

## References

[CR1] Al Aukidy Mustafa, Verlicchi Paola, Voulvoulis Nikolaos (2014). A Framework for the Assessment of the Environmental Risk Posed by Pharmaceuticals Originating from Hospital Effluents. Science of The Total Environment.

[CR2] Annas Julia (2011). Intelligent Virtue.

[CR3] Åström Christofer, Orru Hans, Rocklöv Joacim, Strandberg Gustav, Ebi Kristie L, Forsberg Bertil (2013). Heat-Related Respiratory Hospital Admissions in Europe in a Changing Climate: A Health Impact Assessment. British Medical Journal Open.

[CR4] Beauchamp Tom L, Childress James F (2013). Principles of Biomedical Ethics.

[CR5] Beever Jonathan, Morar Nicolae (2016). The Porosity of Autonomy: Social and Biological Constitution of the Patient in Biomedicine. The American Journal of Bioethics: AJOB.

[CR6] Berkes Fikret, Doubleday Nancy C, Cumming Graeme S (2012). Aldo Leopold’s Land Health from a Resilience Point of View: Self-Renewal Capacity of Social–Ecological Systems. EcoHealth.

[CR7] Bouzid Maha, Colón-González FelipeJ, Lung Tobias, Lake IainR, Hunter PaulR (2014). Climate Change and the Emergence of Vector-Borne Diseases in Europe: Case Study of Dengue Fever. BMC Public Health.

[CR8] British Thoracic Society. 2019. Position Statement on Environment and Lung Health. BTS. https://www.brit-thoracic.org.uk/document-library/audit-and-quality-improvement/environment-and-lung-health/environment-and-lung-health-position-statement-2019/.

[CR9] Buck David (2016). Gardens and Health: Implications for Policy and Practice.

[CR10] Buse Chris G, Smith Maxwell, Silva Diego S (2018). Attending to Scalar Ethical Issues in Emerging Approaches to Environmental Health Research and Practice. Monash Bioethics Review, June.

[CR11] Callahan Daniel (2003). Individual Good and Common Good: A Communitarian Approach to Bioethics. Perspectives in Biology and Medicine.

[CR12] Callicott JBaird, Callicott JBaird (1989). Hume’s Is/Ought Dichotomy and the Relation of Ecology to Leopold’s Land Ethic. In Defense of the Land Ethic.

[CR13] Callicott JBaird, Callicott JBaird (1989). The Conceptual Foundations of the Land Ethic. In Defense of the Land Ethic.

[CR14] Caminade Cyril, Kovats Sari, Rocklov Joacim, Tompkins Adrian M, Morse Andrew P, Colón-González FelipeJ, Stenlund Hans, Martens Pim, Lloyd Simon J (2014). Impact of Climate Change on Global Malaria Distribution. Proceedings of the National Academy of Sciences United States of America.

[CR15] Chung Jeanette W, Meltzer David O (2009). Estimate of the Carbon Footprint of the US Health Care Sector. JAMA.

[CR16] Cohen Aaron J, Brauer Michael, Burnett Richard, Anderson HRoss, Frostad Joseph, Estep Kara, Balakrishnan Kalpana (2017). Estimates and 25-Year Trends of the Global Burden of Disease Attributable to Ambient Air Pollution: An Analysis of Data from the Global Burden of Diseases Study 2015. Lancet.

[CR17] Connor Andrew, Mortimer Frances (2010). The Green Nephrology Survey of Sustainability in Renal Units in England, Scotland and Wales. Journal of Renal Care.

[CR18] Connor Andrew, Mortimer Frances, Tomson Charles (2010). Clinical Transformation: The Key to Green Nephrology. Nephron Clinical Practice.

[CR19] Costello Anthony, Abbas Mustafa, Allen Adriana, Ball Sarah, Bell Sarah, Bellamy Richard, Friel Sharon (2009). Managing the Health Effects of Climate Change. Lancet.

[CR20] Daniels Norman (1979). Wide Reflective Equilibrium and Theory Acceptance in Ethics. The Journal of Philosophy.

[CR21] Davey Peter, Marwick Charis A, Scott Claire L, Charani Esmita, McNeil Kirsty, Brown Erwin, Gould Ian M, Ramsay RCraig, Michie Susan (2017). Interventions to Improve Antibiotic Prescribing Practices for Hospital Inpatients. The Cochrane Library systematic Review.

[CR22] Donker Tjibbe, Wallinga Jacco, Slack Richard, Grundmann Hajo (2012). Hospital Networks and the Dispersal of Hospital-Acquired Pathogens by Patient Transfer. PLoS ONE.

[CR23] Driver Julia (2001). Uneasy Virtue.

[CR24] Gardiner Stephen (2006). A Perfect Moral Storm: Climate Change, Intergenerational Ethics and the Problem of Moral Corruption. Environmental Values.

[CR25] Gillroy John Martin (1998). Kantian Ethics and Environmental Policy Argument: Autonomy, Ecosystem Integrity, and Our Duties to Nature. Ethics and the Environment.

[CR26] Goldberg Tony L, Patz AJonathan (2015). The Need for a Global Health Ethic. The Lancet.

[CR27] Gribble Matthew O’Madigan (2017). Environmental Health Virtue Ethics. The American Journal of Bioethics.

[CR28] Holmes Alison H, Moore Luke SP, Sundsfjord Arnfinn, Steinbakk Martin, Regmi Sadie, Karkey Abhilasha, Guerin Philippe J, Piddock Laura JV (2016). Understanding the Mechanisms and Drivers of Antimicrobial Resistance. The Lancet.

[CR29] Jamieson Dale (2007). When Utilitarians Should Be Virtue Theorists. Utilitas.

[CR30] Kluytmans Jan AJW, Overdevest TMAIlse, Willemsen Ina, van den Bergh Marjolein FQKluytmans, van der Zwaluw Kim, Heck Max (2013). Extended-Spectrum β-Lactamase–Producing Escherichia Coli From Retail Chicken Meat and Humans: Comparison of Strains, Plasmids, Resistance Genes, and Virulence Factors. Clinical Infectious Diseases.

[CR31] Korsgaard Christine (1996). The Sources of Normativity.

[CR32] Korsgaard Christine (2004). Fellow Creatures: Our Obligations to the Other Animals. Uehiro Series in Practical Ethics.

[CR33] Lee Lisa M (2017). A Bridge Back to the Future: Public Health Ethics, Bioethics, and Environmental Ethics. The American Journal of Bioethics.

[CR34] Leopold Aldo (1949). A Sand County Almanac and Sketches Here and There.

[CR35] Little Margaret Olivia (1995). Seeing and Caring: The Role of Affect in Feminist Moral Epistemology. Hypatia.

[CR36] Mackenzie Catriona, Stoljar Natalie (2000). Relational Autonomy: Feminist Perspectives on Autonomy, Agency, and the Social Self.

[CR37] Millstein Roberta L (2018). Debunking Myths about Aldo Leopold’s Land Ethic. Biological Conservation.

[CR38] Myers Samuel S, Wessells KRyan, Kloog Itai, Zanobetti Antonella, Schwartz Joel (2015). Effect of Increased Concentrations of Atmospheric Carbon Dioxide on the Global Threat of Zinc Deficiency: A Modelling Study. The Lancet. Global Health.

[CR39] Naess Arne (1973). The Shallow and the Deep, Long-Range Ecology Movement. Inquiry.

[CR40] NHS SDU (2013). Carbon Footprint Update for the NHS in England 2012.

[CR41] NHS SDU (2016). Carbon Footprint Update for the NHS in England 2015.

[CR42] Noddings Nel (1984). Caring: A Feminine Approach to Ethics and Moral Education.

[CR43] Nozick Robert (1974). Anarchy, State and Utopia.

[CR44] O’Neill, Jim, ed. 2016. Tackling Drug-Resistant Infections Globally: Final Report and Recommendations. The Review on Antimicrobial Resistance. HM Government and the Wellcome Trust.

[CR45] Oaks JLindsay, Gilbert Martin, Virani Munir Z, Watson Richard T, Meteyer Carol U, Rideout Bruce A, Shivaprasad HL (2004). Diclofenac Residues as the Cause of Vulture Population Decline in Pakistan. Nature.

[CR46] Paltansing Sunita, Vlot Jessica A, Kraakman Margriet EM, Mesman Romy, Bruijning Marguerite L, Bernards Alexandra T, Visser Leo G, Veldkamp Karin Ellen (2013). Extended-Spectrum β-Lactamase-Producing Enterobacteriaceae among Travelers from the Netherlands. Emerging Infectious Diseases.

[CR47] Parfit Derek (1984). Reasons and Persons.

[CR48] Pellegrino Edmund D, Bulger Roger J (1973). Toward an Expanded Medical Ethics: The Hippocratic Ethic Revisited. Hippocrates Revisited.

[CR49] Pellegrino Edmund D (2006). Toward a Reconstruction of Medical Morality. The American Journal of Bioethics: AJOB.

[CR50] Potter Van Rensselaer (1971). Bioethics: Bridge to the Future.

[CR51] Potter Van Rensselaer (1988). Global Bioethics: Building on the Leopold Legacy.

[CR52] Regan Tom (2004). The Case for Animal Rights.

[CR53] Reich Warren Thomas (1995). The Word ‘Bioethics’: The Struggle over Its Earliest Meanings. Kennedy Institute of Ethics Journal.

[CR54] Resnik David B (2012). Environmental Health Ethics.

[CR55] Robinson TP, Bu DP, Carrique-Mas J, Fèvre EM, Gilbert M, Grace D, Hay SI (2016). Antibiotic Resistance Is the Quintessential One Health Issue. Transactions of The Royal Society of Tropical Medicine and Hygiene.

[CR56] Schlosberg David, Collins Lisette B (2014). From Environmental to Climate Justice: Climate Change and the Discourse of Environmental Justice. Wiley Interdisciplinary Reviews: Climate Change.

[CR57] Sherwin Susan, Downie Jocelyn, Llewellyn Jennifer J (2011). Relational Autonomy and Global Health Threats. Being Relational: Reflections on Relational Theory and Health Law.

[CR58] Shrader-Frechette Kristin (2002). Environmental Justice: Creating Equality, Reclaiming Democracy.

[CR59] Singer Peter, Beauchamp Tom L, Regan Tom (1980). Animals and the Value of Life. Matters of Life and Death.

[CR60] Spurling Geoffrey KP, Chris B, Del Mar Liz, Dooley Ruth, Foxlee, Farley Rebecca (2017). Delayed Antibiotic Prescriptions for Respiratory Infections. Cochrane Database of Systematic Reviews.

[CR61] Stephenson Judith, Crane Susan F, Levy Caren, Maslin Mark (2013). Population, Development, and Climate Change: Links and Effects on Human Health. The Lancet.

[CR62] Svoboda Toby (2012). Duties Regarding Nature: A Kantian Approach to Environmental Ethics. Kant Yearbook.

[CR63] Syed Shamsuzzoha B, Dadwal Viva, Rutter Paul, Storr Julie, Hightower Joyce D, Gooden Rachel, Carlet Jean (2012). Developed-Developing Country Partnerships: Benefits to Developed Countries?. Globalization and Health.

[CR64] Taylor Charles (1991). The Malaise of Modernity.

[CR65] ten Have Henk AMJ (2012). Potter’s Notion of Bioethics. Kennedy Institute of Ethics Journal.

[CR66] Verweij Marcel, Bovenkerk Bernice (2016). Ethical Promises and Pitfalls of OneHealth. Public Health Ethics.

[CR67] WHO (2017). Climate Change and Health Factsheet.

[CR68] Wynia Matthew K (2008). The Short History and Tenuous Future of Medical Professionalism: The Erosion of Medicine’s Social Contract. Perspectives in Biology and Medicine.

